# A Novel Digital Digit-Symbol Substitution Test Measuring Processing Speed in Adults At Risk for Alzheimer Disease: Validation Study

**DOI:** 10.2196/36663

**Published:** 2023-01-27

**Authors:** Anthony Campitelli, Sally Paulson, Josh L Gills, Megan D Jones, Erica N Madero, Jennifer Myers, Jordan M Glenn, Michelle Gray

**Affiliations:** 1 Exercise Science Research Center University of Arkansas Fayetteville, AR United States; 2 Neurotrack Technologies, Inc Redwood City, CA United States

**Keywords:** Alzheimer disease, dementia, processing speed, digit-symbol substitution, aging, cognitive

## Abstract

**Background:**

Assessing cognitive constructs affected by Alzheimer disease, such as processing speed (PS), is important to screen for potential disease and allow for early detection. Digital PS assessments have been developed to provide widespread, efficient cognitive testing, but all have been validated only based on the correlation between test scores. Best statistical practices dictate that concurrent validity should be assessed for agreement or equivalence rather than using correlation alone.

**Objective:**

This study aimed to assess the concurrent validity of a novel digital PS assessment against a gold-standard measure of PS.

**Methods:**

Adults aged 45-75 years (n=191) participated in this study. Participants completed the novel digital digit-symbol substitution test (DDSST) and the Repeatable Battery for the Assessment of Neuropsychological Status coding test (RBANS-C). The correlation between the test scores was determined using a Pearson product-moment correlation, and a difference in mean test scores between tests was checked for using a 2-tailed dependent samples *t* test. Data were analyzed for agreement between the 2 tests using Bland-Altman limits of agreement and equivalency using a two one-sided *t* tests (TOST) approach.

**Results:**

A significant moderate, positive correlation was found between DDSST and RBANS-C scores (*r*=.577; *P*<.001), and no difference in mean scores was detected between the tests (*P*=.93). Bias was nearly zero (0.04). Scores between the tests were found to display adequate agreement with 90% of score differences falling between –22.66 and 22.75 (90% limits of agreement=–22.91 to 22.99), and the scores were equivalent (*P*=.049).

**Conclusions:**

Analyses indicate that the DDSST is a valid digital assessment of PS. The DDSST appears to be a suitable option for widespread, immediate, and efficient PS testing.

**Trial Registration:**

ClinicalTrials.gov NCT04559789; https://clinicaltrials.gov/ct2/show/NCT04559789

## Introduction

Currently, Alzheimer disease (AD) affects more than 6 million Americans, and by the year 2050, this number is expected to rise to more than 13 million [1]. One in 3 seniors will die while experiencing AD or a related dementia, and the yearly deaths directly attributable to AD account for more than breast and prostate cancer combined [1]. AD and associated dementias also incur a substantial economic and social cost. It is estimated that in 2021, these cognitive diseases cost Americans US $355 billion; this figure is projected to rise to US $1.1 trillion by 2050. Socially, in 2020, more than 11 million unpaid caregivers worked 15.3 billion hours providing support for individuals with AD and related dementias—their time is valued at US $257 billion in lost wages [1]. Recognizing this dismal outlook, the research community has investigated many of the cognitive constructs associated with the disease in an effort to better understand the progression of the illness, detect signs earlier, and potentially develop methods to mitigate it [2].

One cognitive construct researchers have investigated in the context of AD is processing speed (PS). PS is defined as the rate at which an individual can analyze cognitive stimuli and complete cognitive tasks [3,4], and it has been shown to decrease significantly in individuals with mild cognitive impairment and further still in individuals with AD [3]. As such, PS is an important component in cognitive assessment protocols and AD-monitoring programs [5]. Several laboratory- and clinic-based assessments of PS have been developed [5], but the limited availability of these tests concerns researchers as neurobiological decline can occur 15 years before any cognitive deficits are measurable, indicating that testing should take place frequently and ubiquitously to ensure that cognitive decline is detected as early as possible [2,6,7]. More recently, to address the need for widespread, rapid testing, researchers have focused on the development of digital cognitive tests that can be taken quickly on a mobile device and demonstrate high scalability, efficiency, and convenience [6,8]. Although several digital PS tests have been developed and presented as valid assessments [9-11], the validation procedures followed in these studies only examined the linear relationship between novel test scores and a gold-standard test, not the *agreement* between individual scores as is required for proper validation [12]. The purpose of this study was to validate a novel PS task, a digital digit-symbol substitution test (DDSST), by examining its concurrent validity through comparison to a gold-standard test of PS. As tests of digit-symbol coding have been identified as gold-standard measures of PS [13], the Repeatable Battery for the Assessment of Neuropsychological Status (RBANS) coding test (RBANS-C) [14,15] was chosen as a comparison for its validity in the population analyzed in this study [15].

## Methods

### Study Design

This study implemented a cross-sectional design to evaluate concurrent validity between instruments. As a validation study, no random assignment was possible or required. Additionally, no form of blinding was used in this study design.

### Participants

The goal sample size for this study was set at 200 participants to ensure adequate statistical power while allowing for potential attrition and incomplete data. Criteria for inclusion were adults aged between 45 and 75 years, BMI between 18.5 and 39.9 kg/m^2^, and at least two of the following Alzheimer risk factors: high school education or less; BMI >25; and history of diabetes, hypertension, high cholesterol, or smoking. Participants were excluded if they had a diagnosed mental health condition, dementia, probable dementia, mild cognitive impairment, or other major health condition; a recent cardiovascular event; vision problems that would prevent viewing of a screen; learning disability; or more than one of the following Alzheimer protective factors: a high level of physical activity, a high level of fish consumption, or a high level of cognitive engagement. Participants were recruited in northwest Arkansas using local radio, emails, social media advertisements, news releases, and word of mouth.

### Procedure

Participants reported to the laboratory and completed the RBANS assessment with an experienced test administrator. RBANS assessment procedures are described in detail elsewhere [14], but briefly, the RBANS-C assessment asks participants to match sequential symbols with corresponding numbers from a key on the same page, writing the correct number below each symbol. The RBANS-C raw score is calculated as the number of correct numbers filled in within 90 seconds. Assessments were scored by an experienced test administrator in accordance with the RBANS Testing Manual [14].

**Figure 1:**
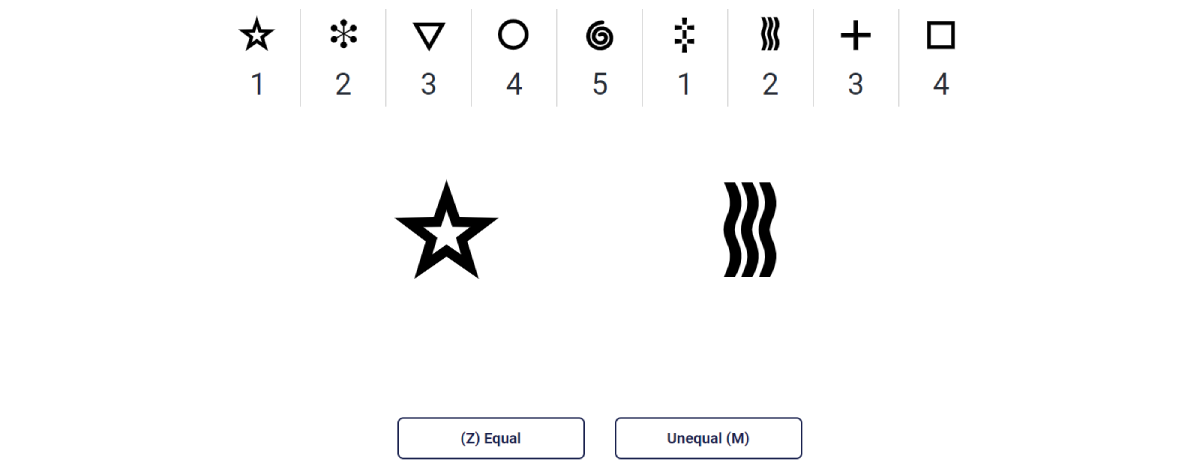
DDSST testing screen seen by participants. In this example, the correct response would be that the star (value of 1) and wavy lines (value of 2) do not represent equal values based on the key. The participant would press the “M” key on their keyboard in this case. DDSST: digital digit-symbol substitution test.

After a minimum of 30 minutes of unrelated physical testing, participants were instructed to begin using the self-guided cognitive testing platform (Neurotrack Digital Testing Platform; Neurotrack Technologies Inc) containing the DDSST test. The DDSST is based on the Digit Symbol Substitution Test [16]. The assessment provided both written and visual step-by-step, on-screen instructions; asked participants to determine if 2 symbols were equal or unequal based on a legend with 9 number-symbol pairs (Figure 1); and was objectively scored based on accuracy and speed using Neurotrack’s automated scoring algorithm. Specifically, raw scores were calculated as the number of correct responses given in 2 minutes. Data for this study were collected as part of an ongoing parent study (Digital Cognitive Multi-domain Alzheimer’s Risk Velocity [DC MARVel] Study) that is longitudinally examining changes in cognition and AD risk measures in at-risk adults randomized into either a health education or health-coaching intervention [17]. Further information regarding other subtests found in the Neurotrack Digital Testing Platform can be found in the published protocol for the DC MARVel Study [17].

### Data Analysis

All statistical analyses were performed utilizing the *Analyse-It* extension for Microsoft Excel. The linear relationship between the RBANS-C and DDSST raw scores was calculated using a Pearson product-moment correlation. For tests of mean differences, agreement, and equivalency, the raw scores for both tests were scaled to a distribution with a mean of 100 and an SD of 15 to allow for the direct comparison of scores without impacting the distribution of scores or the appropriateness of the tests being used. In the case of scaling the RBANS-C score, the obtained score was converted to a *z* score based on the accepted population mean and SD for RBANS-C raw scores and then multiplied by 15, but as no accepted DDSST population mean and SD exists, those obtained from this study were used.

Differences between group means were assessed using a 2-tailed dependent samples *t* test. Agreement was assessed with a Bland-Altman limits of agreement (LoA) plot with a 90% LoA. The LoA cutoff was set at the mean bias plus or minus the minimum reliable change (RC) score for the RBANS-C test obtained from previous literature (*z*=1.53) [12]. Researchers have stressed that RC is a more appropriate metric for assessing minimum change in the RBANS test than the minimum clinically important difference or standard error of measure [18]. A mountain plot was also generated to assess the y-axis distribution of the mean-difference plot.

Equivalency was tested using a two one-sided *t* tests (TOST) analysis, and the equivalency upper and lower bounds were determined [19] using the RBANS RC score obtained from previous literature described above. An a priori α level of .05 was used for all appropriate analyses.

### Ethics Approval

This study was approved by the Institutional Review Board at the University of Arkansas (protocol #2009280813).

### Informed Consent

Participants were informed of their rights as research participants and clearly notified that their participation was voluntary and they could withdraw at any time. All participants signed an approved consent form in accordance with the ethical standards of Helsinki.

## Results

### Participants

In total, 210 participants were initially enrolled in this study. After prestudy attrition and adjusting the data set for incomplete testing data, a final sample of 191 adults (female: n=138; male: n=53) was analyzed. The average age of the sample was 62.2 (SD 8.27) years. Further descriptive statistics are presented in Table 1.

**Table 1:** Participant descriptive statistics (n=191).

Measure	Value, mean (SD)	95% CI
Age (years)	62.2 (8.27)	63.4-61.0
Height (cm)	167.5 (9.13)	168.8-166.2
Weight (kg)	84.9 (18.23)	87.5-82.3
BMI (kg/m^2^)	30.1 (5.22)	30.9-29.4
RBANS-C^a^ score^b^	49.4 (8.09)	50.6-48.3
DDSST^c^ score^b^	26.1 (6.57)	27.1-25.2

^a^RBANS-C: Repeatable Battery for the Assessment of Neuropsychological Status coding test.

^b^RBANS-C and DDSST scores are presented as raw scores here.

^c^DDSST: digital digit-symbol substitution test.

### Data Analysis

**Figure 2:**
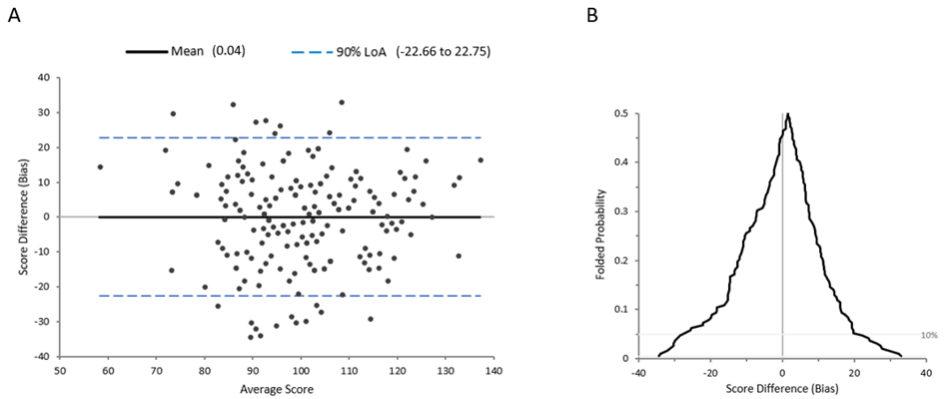
Bland-Altman plot (A) and mountain plot (B) comparing the novel DDSST test to RBANS coding test. DDSST: digital digit-symbol substitution test; LoA: limits of agreement; RBANS: Repeatable Battery for the Assessment of Neuropsychological Status.

The moderately positive correlation obtained for the RBANS-C raw score and DDSST raw score was statistically significant (*r*=.577; *P*<.001). A 2-tailed dependent-samples *t* test showed no significant differences between the RBANS-C and DDSST scores within participants (*t*_190_=–0.09; *P*=.93). Bland-Altman plots revealed the 90% LoA (–22.66 to 22.75) was within the a priori cutoff (–22.91 to 22.99), indicating that the scores for RBANS-C and DDSST were in acceptable agreement. Additionally, the mean bias score was near zero (0.04), indicating low systemic bias in scores, and there was no obvious linear pattern in the scatter plot distribution (Figure 2). The mountain plot distribution was roughly symmetrical, had a peak close to zero bias, and had no obvious tail skew (Figure 2). TOST analysis indicated that the scores for RBANS-C and DDSST were equivalent.

## Discussion

### Principal Findings

The purpose of this study was to assess the concurrent validity of a novel DDSST test as compared to the RBANS-C, a gold-standard measure of PS. Results showed no statistically significant differences in mean test scores within participants; a significant, moderately positive correlation in individual test scores; and acceptable agreement and equivalency between the 2 assessments. These results indicate that the DDSST is a valid assessment tool for evaluating PS in the sample of middle-age and older adults.

### Impact

The potential impact of an assessment such as the DDSST is greater when the efficiency, scalability, and convenience of a digitally enabled test is considered. Digital tests allow the instant reporting of results to the test taker and potentially clinicians; the immediate and ad hoc distribution of tests to any number of test takers; and the convenience of having the ability to take tests anywhere, at any time.

Compared to previous studies validating digital assessments of PS, the relationship between the novel test and gold-standard test was lower here (*r*=.577 vs *r*=.75-.8) [10,11]. Validation, however, should not be based upon correlation between scores as this does not accurately demonstrate agreement between individual scoring pairs but rather an overall linear relationship that minimizes residuals [12]. Of the previous studies of digital instruments evaluating PS reviewed by the authors, none analyzed or reported the agreement between paired measures from novel and gold-standard tests in their assessment of concurrent validity [9-11]. As such, this study conducted a more comprehensive analysis of concurrent validity through the additional examination of agreement and appears to be the first study of a digital PS test to base its validation on those criteria.

### Limitations

First, this study may have been limited by its protocol. It was carried out as part of a larger trial, and the order of testing was not randomized in the protocol to minimize learning effects or cognitive fatigue effects. Second, the sampling procedure specifically excluded individuals with cognitive impairments. By excluding segments that exist in the general population, generalizability is reduced. Third, intrarater and test-retest reliability could not be assessed for the novel DDSST instrument as repeat trials were not conducted as a part of this data set.

### Future Directions

Future studies should include individuals with cognitive impairments to assess validity in a broader population segment, as well as assess the ability of the DDSST to discriminate between levels of cognition. Although the test-retest reliability of the DDSST has been verified in a different sample [20], additional psychometric testing with a representative population is warranted to ensure the full utility of the measure.

### Conclusion

Despite the study’s limitations, the DDSST shows promising clinical utility. DDSST scores were in agreement with, and equivalent to, scores obtained from the RBANS-C, a gold-standard test of PS. Given its demonstrated concurrent validity, the DDSST appears to be a suitable option for widespread and rapid cognition testing. Further investigation, however, is required to assess the reliability of the instrument.

## Abbreviations

AD: Alzheimer disease
DC MARVel: Digital Cognitive Multi-domain Alzheimer’s Risk Velocity
DDSST: digital digit-symbol substitution test
LoA: limits of agreement
PS: processing speed
RBANS: Repeatable Battery for the Assessment of Neuropsychological Status
RBANS-C: Repeatable Battery for the Assessment of Neuropsychological Status coding test
RC: reliable change
TOST: two one-sided *t* tests

## Data Availability

The data sets generated or analyzed during the current study are not publicly available due to the ongoing nature of the parent study, but aggregate data specific to the present study are available from the corresponding author on reasonable request.

## References

[1] (2022). 2022 Alzheimer's disease facts and figures. Alzheimer's Association.

[2] de Roeck EE, de Deyn PP, Dierckx E, Engelborghs S (2019). Brief cognitive screening instruments for early detection of Alzheimer's disease: a systematic review. Alzheimers Res Ther.

[3] Bublak P, Redel P, Sorg C, Kurz A, Förstl Hans, Müller Hermann J, Schneider WX, Finke K (2011). Staged decline of visual processing capacity in mild cognitive impairment and Alzheimer's disease. Neurobiol Aging.

[4] Kail R, Salthouse TA (1994). Processing speed as a mental capacity. Acta Psychol (Amst).

[5] Salthouse TA (2000). Aging and measures of processing speed. Biol Psychol.

[6] Bott N, Madero EN, Glenn J, Lange A, Anderson J, Newton D, Brennan A, Buffalo EA, Rentz D, Zola S (2018). Device-embedded cameras for eye tracking–based cognitive assessment: validation with paper-pencil and computerized cognitive composites. J Med Internet Res.

[7] Kremen William S, Jak Amy J, Panizzon Matthew S, Spoon Kelly M, Franz Carol E, Thompson Wesley K, Jacobson Kristen C, Vasilopoulos Terrie, Vuoksimaa Eero, Xian Hong, Toomey Rosemary, Lyons Michael J (2014). Early identification and heritability of mild cognitive impairment. Int J Epidemiol.

[8] Gills JL, Glenn JM, Madero EN, Bott NT, Gray M (2019). Validation of a digitally delivered visual paired comparison task: reliability and convergent validity with established cognitive tests. Geroscience.

[9] Weintraub S, Dikmen SS, Heaton RK, Tulsky DS, Zelazo PD, Slotkin J, Carlozzi NE, Bauer PJ, Wallner-Allen K, Fox N, Havlik R, Beaumont JL, Mungas D, Manly JJ, Moy C, Conway K, Edwards E, Nowinski CJ, Gershon R (2014). The cognition battery of the NIH toolbox for assessment of neurological and behavioral function: validation in an adult sample. J Int Neuropsychol Soc.

[10] Rao SM, Losinski G, Mourany L, Schindler D, Mamone B, Reece C, Kemeny D, Narayanan S, Miller DM, Bethoux F, Bermel RA, Rudick R, Alberts J (2017). Processing speed test: validation of a self-administered, iPad-based tool for screening cognitive dysfunction in a clinic setting. Mult Scler.

[11] Gorodeski EZ, Rosenfeldt AB, Fang K, Kubu C, Rao SM, Jansen EA, Dey T, Alberts JL (2019). An iPad-based measure of processing speed in older adults hospitalized for heart failure. J Cardiovasc Nurs.

[12] Bland JM, Altman DG (1999). Measuring agreement in method comparison studies. Stat Methods Med Res.

[13] Carlozzi NE, Tulsky DS, Chiaravalloti ND, Beaumont JL, Weintraub S, Conway K, Gershon RC (2014). NIH Toolbox Cognitive Battery (NIHTB-CB): the NIHTB pattern comparison processing speed test. J Int Neuropsychol Soc.

[14] Randolph C (1998). Repeatable Battery for the Assessment of Neuropsychological Status (RBANS).

[15] Randolph C, Tierney MC, Mohr E, Chase TN (1998). The Repeatable Battery for the Assessment of Neuropsychological Status (RBANS): preliminary clinical validity. J Clin Exp Neuropsychol.

[16] Jaeger J (2018). Digit symbol substitution test: the case for sensitivity over specificity in neuropsychological testing. J Clin Psychopharmacol.

[17] Gray M, Madero EN, Gills J, Paulson S, Jones MD, Campitelli A, Myers Jennifer, Bott Nicholas T, Glenn Jordan M (2022). Intervention for a digital, cognitive, multi-domain Alzheimer risk velocity study: protocol for a randomized controlled trial. JMIR Res Protoc.

[18] O'Connell Megan E, Gould B, Ursenbach J, Enright J, Morgan DG (2019). Reliable change and minimum clinically important difference (MCID) of the Repeatable Battery for the Assessment of Neuropsychology Status (RBANS) in a heterogeneous dementia sample: support for reliable change methods but not the MCID. Appl Neuropsychol Adult.

[19] Lakens D (2017). Equivalence tests: a practical primer for tests, correlations, and meta-analyses. Soc Psychol Personal Sci.

[20] Myers JR, Glenn JM, Madero EN, Anderson J, Mak-McCully R, Gray M, Gills JL, Harrison JE (2022). Asynchronous remote assessment for cognitive impairment: reliability verification of the Neurotrack Cognitive Battery. JMIR Form Res.

